# Overexpression of topoisomerase II alpha protein is a factor for poor prognosis in patients with luminal B breast cancer

**DOI:** 10.18632/oncotarget.25468

**Published:** 2018-06-01

**Authors:** Hideo Shigematsu, Shinji Ozaki, Daisuke Yasui, Hideki Yamamoto, Junichi Zaitsu, Daiki Taniyama, Akihisa Saitou, Kazuya Kuraoka, Taizo Hirata, Kiyomi Taniyama

**Affiliations:** ^1^ Department of Breast Surgery, National Hospital Organization Kure Medical Center and Chugoku Cancer Center, Kure-City, Hiroshima, Japan; ^2^ Department of Pathology, National Hospital Organization Kure Medical Center and Chugoku Cancer Center, Kure-City, Hiroshima, Japan; ^3^ Department of Medical Oncology, National Hospital Organization Kure Medical Center and Chugoku Cancer Center, Kure-City, Hiroshima, Japan; ^4^ National Hospital Organization Kure Medical Center and Chugoku Cancer Center, Kure-City, Hiroshima, Japan

**Keywords:** breast cancer, topoisomerase IIA, prognostic value, luminal B

## Abstract

**Background:**

The prognostic value and the best method of testing of topoisomerase II alpha (TOP2A) status have not been established in modern tailored therapy based on breast cancer subtype.

**Results:**

The frequencies of TOP2A overexpression and *TOP2A* amplified were 55.8% and 9.5%, respectively. TOP2A overexpression correlated strongly with non-luminal A subtype (χ^2^-test, *p <* 0.001). TOP2A overexpression was significantly associated with relapse-free survival in luminal B breast cancer (*n* = 316; log rank test, *p <* 0.001) but not in other breast cancer subtypes. Cox regression analysis showed that TOP2A overexpression is a significant prognostic factor in luminal B breast cancer (hazard ratio (HR) 4.00, 95% confidence interval (CI) 1.65–9.54, *p* = 0.002). *TOP2A* amplified was recognized in HER2 positive breast cancer (*p <* 0.001). In HER2 positive breast cancer, *TOP2A* amplified (HR 0.30, 95% CI 0.085–1.07, *p* = 0.063) appeared to be a better prognostic factor.

**Conclusion:**

In modern tailored therapy, TOP2A overexpression can be a poor prognostic factor in luminal B breast cancer. In contrast, *TOP2A* amplified could be a better prognostic factor in HER2 positive breast cancer.

**Materials and methods:**

Between May 2005 and April 2015, a total of 643 consecutive non-metastatic invasive breast cancers were evaluated for *TOP2A* amplified using fluorescence *in situ* hybridization analysis (FISH) and for TOP2A overexpression using the immunohistochemistry assay. FISH ratios of 2 or higher were designated as *TOP2A* amplified, and TOP2A staining >10% was defined as TOP2A overexpression. The prognostic values of *TOP2A* amplified and TOP2A overexpression were retrospectively evaluated.

## INTRODUCTION

Topoisomerase IIA (TOP2A) is an essential nuclear enzyme that cleaves and recombines double-stranded DNA in processes such as replication, transcription, condensation, and segregation [1]. TOP2A is expressed in proliferating cells in the late S and the G2-M phases, and TOP2A expression is known to be a prognostic factor in various malignancies [2–5]. In breast cancer, TOP2A protein or gene expression is also associated with a poor prognosis [6, 7]; the prognostic value is emphasized in hormone receptor-positive disease [8, 9]. Additionally, TOP2A is a molecular target of anthracycline, and previous reports have shown that *TOP2A* amplified or deleted are predictive markers of anthracycline-containing adjuvant chemotherapy regimens for early breast cancer [10–13].

Although TOP2A status has several important implications in breast cancer, the standard tools and cutoff values for estimating TOP2A status have not been established [14]. Additionally, previous reports of the predictive and prognostic value of TOP2A status were based on classical adjuvant chemotherapy regimens without taxane and HER2-targeting therapies, which differed from modern clinical practice. The clinical significance and best assessment of TOP2A status need to be re-evaluated under modern tailored therapy based on breast cancer subtype.

At our institution, both TOP2A overexpression and *TOP2A* amplified had been consecutively evaluated using immunohistochemistry (IHC) and fluorescence *in situ* hybridization (FISH) in patients with breast cancer between May 2005 and April 2015. During this period, adjuvant therapies, including taxane and HER2 targeting therapy, were administered based on breast cancer subtype and the risk for recurrence. We retrospectively evaluated the prognostic value of TOP2A status in these patients with non-metastatic breast cancer.

## RESULTS

### Patient characteristics and TOP2A status

The median and mean of TOP2A expression were 11.3% and 18.4%, respectively. Histogram of continuous IHC proportion scores of TOP2A is shown in (Supplementary Figure 3). The frequency of TOP2A overexpression was 55.8% (359/643). The numbers for *TOP2A* amplified, normal, and deleted were 61 (9.5%), 577 (89.7%), and 5 (0.8%), respectively. The associations of the clinicopathological factors with TOP2A overexpression and *TOP2A* amplified are shown in Tables 1 and 2, respectively. TOP2A overexpression showed a significant correlation with larger T stage (*p <* 0.001), node positivity (*p <* 0.001), high nuclear grade (*p <* 0.001), ER negativity (*p <* 0.001), PgR negativity (*p <* 0.001), HER2 positivity (*p <* 0.001), and high Ki67 index (*p <* 0.001) using the χ^2^-test. TOP2A overexpression was recognized in non-luminal A subtype (rate of overexpression, luminal A vs. non-luminal A, 8.3% vs. 65.4%, χ^2^-test, *p <* 0.001). Although the associations between *TOP2A* amplified and several clinicopathological factors, including larger T, high nuclear grade, and high Ki67 index, were significant, almost *TOP2A* amplified was observed in HER2 positive breast cancers; specifically, the rates of *TOP2A* amplified were 42.2% (57/135) and 0.8% (4/508) in HER2-positive and -negative cancers, respectively. Thus, the characteristics of *TOP2A* amplified breast cancer reflected those of HER2 positive breast cancer.

**Table 1:** Clinicopathological factors and TOP2A overexpression

			TOP2A overexpression				
factor		total	yes	%	no	%	*p* value
		643	359	55.8	284	44.2	
age	50≥	14	87	61.7	54	38.3	0.11
	50<	502	272	54.2	230	45.8	
T	^*^0	2	2	100	0	0	<0.001
	1	366	163	44.5	203	55.5	
	2	211	143	32.2	68	67.8	
	3	36	26	72.2	10	27.8	
	4	28	25	89.3	3	10.7	
N	0	422	211	50	211	50	<0.001
	1	172	111	64.5	61	35.5	
	2	35	27	77.1	8	22.9	
	3	14	4	71.4	10	28.6	
Stage	1	302	132	43.7	170	56.3	<0.001
	2	262	166	63.4	96	33.8	
	3	79	61	77.2	18	22.8	
nuclear grade	1	291	83	28.5	208	71.5	<0.001
	2	161	103	64	58	36	
	3	263.6	173	90.6	18	9.4	
ER	positive	498	250	50.2	248	49.8	<0.001
	negative	145	109	75.2	36	24.8	
PgR	positive	434	218	50.2	216	49.8	<0.001
	negative	209	141	67.5	68	32.5	
HER2	positive	134	107	79.3	27	20.7	<0.001
	negative	509	252	49.6	257	50.4	
Ki 67	14%>	211	23	10.9	188	89.1	<0.001
	≧14%	432	336	77.8	96	22.2	
subtype	luminal A	108	9	8.3	99	91.7	<0.001
	luminal B	315	179	56.8	136	43.2	
	HER2	135	107	79.3	28	20.7	
	TNBC	85	64	75.3	21	24.7	
TOP2A amplified	yes	61	48	78.7	13	21.3	<0.001
	no	582	311	53.4	271	46.6	
Adjuvant chemotherapy	yes	309	235	76.1	74	23.9	<0.001
	no	334	124	37.1	210	62.9	
Adjuvant radiation therapy	yes	413	232	56.2	181	44.2	0.82
	no	230	127	55.2	103	44.8	
recurrence	yes	73	60	82.2	13	17.8	<0.001
	no	570	299	52.5	271	47.5	

*p* value was evaluated using χ^2^ test, ^*^T0 means occult breast cancer.

Abbreviations: ER: estrogen receptor; PgR; progesteron receptor; HER2: human epidermal growth factor receptor 2 TNBC: triple negative breast cancer.

**Table 2:** Clinicopathological factors and *TOP2A* amplified

			*TOP2A* amplified				
factor		total	yes	%	no	%	*p* value
		643	61	9.5	582	90.5	
age	50≥	14	17	12.1	124	87.9	0.24
	50<	502	44	8.8	458	90.2	
T	^*^0	2	0	0	2	100	0.044
	1	366	28	7.7	338	92.3	
	2	211	22	10.4	189	89.6	
	3	36	4	11.1	32	88.9	
	4	28	7	25	21	75	
N	0	422	37	8.8	385	91.2	0.052
	1	172	15	8.7	157	91.3	
	2	35	8	22.9	27	77.1	
	3	14	1	7.1	13	92.9	
Stage	1	302	27	8.9	275	91.1	0.073
	2	262	21	8	241	92	
	3	79	13	16.5	66	83.5	
nuclear grade	1	291	12	4.1	279	95.9	<0.001
	2	161	19	11.8	142	88.2	
	3	263.6	30	15.7	161	84.3	
ER	positive	498	41	8.2	457	91.8	0.044
	negative	145	20	13.8	125	86.2	
PgR	positive	434	39	9	395	91	0.48
	negative	209	22	10.5	187	89.5	
HER2	positive	134	57	42.2	78	57.8	<0.001
	negative	509	4	0.8	504	99.2	
Ki 67	14%>	211	13	6.2	198	93.8	0.044
	≧14%	432	48	10.9	384	89.1	
subtype	luminal A	108	0	0	108	100	<0.001
	luminal B	315	1	0.3	314	99.7	
	HER2	135	57	42.2	78	57.8	
	TNBC	85	3	3.5	82	96.5	
TOP2A overexpression	yes	359	48	13.3	311	86.7	<0.001
	no	284	13	4.6	271	95.4	
Adjuvant chemotherapy	yes	309	48	15.5	261	84.5	<0.001
	no	334	13	3.9	321	96.1	
Adjuvant radiation therapy	yes	413	41	9.9	372	90.1	0.61
	no	230	20	8.7	210	91.3	
recurrence	yes	73	4	5.5	69	94.5	0.21
	no	570	57	10	513	90	

*p* value was evaluated using χ^2^ test, ^*^T0 means occult breast cancer.

Abbreviations: ER: estrogen receptor; PgR; progesteron receptor; HER2: human epidermal growth factor receptor 2 TNBC: triple negative breast cancer.

### Prognostic value of TOP2A overexpression in each breast cancer subtype

**Figure 1:**
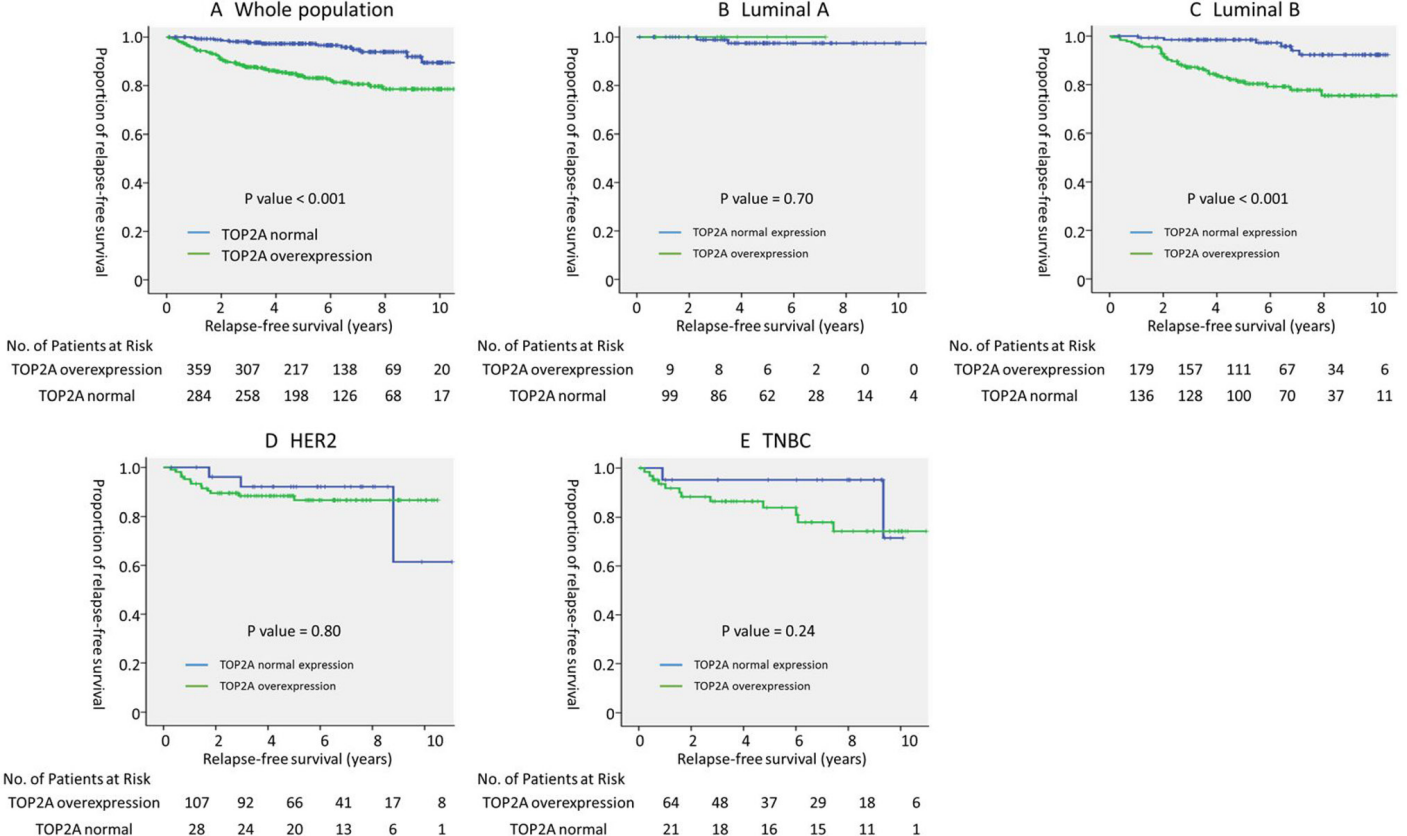
Prognostic value of TOP2A overexpression in each breast cancer subtype Relapse-free survival of (**A**) all (*n* = 643), (**B**) luminal A (*n* = 108), (**C**) luminal B (*n* = 315), (**D**) HER2 (*n* = 135), and (**E**) TNBC (*n* = 85) subtypes stratified by TOP2A overexpression. *P* value was evaluated using the log-rank test. Abbreviations: HER2: human epidermal growth factor receptor 2; TNBC: triple negative breast cancer.

In this study, the median follow-up period was 5.4 years. In the entire population, there was a significant association between TOP2A overexpression and relapse-free survival (5-year RFS; TOP2A overexpression vs. normal expression, 97.3% vs. 84.1%, *p <* 0.001, log-rank test) (Figure 1A).

In the subgroup analysis of breast cancer subtypes, TOP2A overexpression was a significant prognostic factor in luminal B breast cancer (5-year RFS; TOP2A overexpression vs. no overexpression, 98.5% vs. 81.3%, *p <* 0.001, log-rank test); however, no such significant association was observed in other subtypes (Figure 1B–1E). In luminal B breast cancer, Cox regression analysis that included other prognostic factors, such as, age, T factor, N factor, nuclear grade, PgR positivity, and Ki67 index, showed that TOP2A overexpression was the strongest prognostic factor for RFS (hazard ratio [HR] 4.00, 95% confidence interval [CI] 1.65–9.54, *p <* 0.001) (Table 3). In an exploratory analysis, receiver operating characteristic (ROC) curve of SUVmax was drawn to determine the cutoff value that yielded optimal sensitivity and specificity for prediction of 5-year RFS in luminal B breast cancer based on the Youden index. The ROC curve indicated an optimal TOP2A expression cutoff value of 11.5% for predicting 5-year RFS (area under the curve, 0.74; 95% CI, 0.66–0.82; *P* < .001; sensitivity, 87.9%; specificity, 59.6%). A continuous value of TOP2A expression was also shown to be the significant prognostic factor in luminal B breast cancer in Cox regression analysis (HR 1.07; 95% CI, 1.02–1.12, *P* = 0.005).

**Table 3:** Multivariate Cox regression analysis of clinicopathological factors and TOP2A overexpression, with respect to relapse-free survival among patients with luminal B breast cancer (*n =* 315)

factors		*n*	hazard ratio (95% CI)	*p* value
age	50≥	69	1	0.30
	50<	246	0.68 (0.34–1.36)	
T stage	2 cm≥	177	1	0.036
	2 cm<	138	2.15 (1.05–4.39)	
Nodal status	netative	202	1	0.012
	positive	113	2.37 (1.21–4.62)	
PgR	positive	249	1	0.57
	netative	66	1.26 (0.57–2.78)	
nuclear grade	1, 2	236	1	0.85
	3	79	1.1 (0.55–2.30)	
Ki67 (continuous value)			1.02 (1.00–1.04)	0.11
adjuvant chemotherapy	no	183	1	0.76
	yes	132	0.89 (0.42–1.88)	
adjuvant radiation therapy	no	105	1	0.29
	yes	210	0.71 (0.31–1.35)	
TOP2A overexpression	no	136	1	0.002
	yes	179	4.00 (1.65–9.54)	

Abbreviations: PgR; progesteron receptor.

### Prognostic value of *TOP2A* amplified in HER2 positive breast cancer

**Figure 2:**
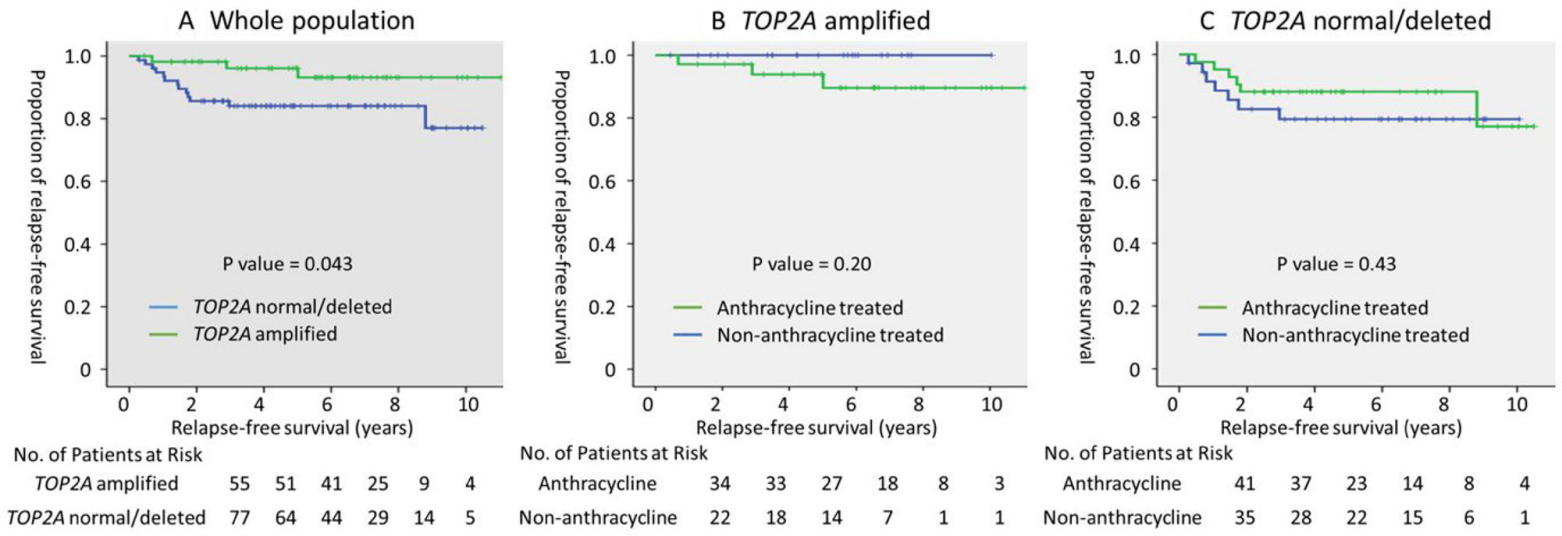
Prognostic value of *TOP2A* amplified and adjuvant anthracycline therapy in HER2 positive breast cancer (**A**) Relapse-free survival of the whole population (*n* = 135), and the (**B**) *TOP2A* amplified (*n* = 57), and (**C**) *TOP2A* normal/deleted (*n* = 78) populations, stratified by adjuvant anthracycline treatment. *P* value was evaluated using the log-rank test.

Because *TOP2A* amplified was strongly associated with HER2 positivity, the prognostic value of *TOP2A* amplified was examined using the log-rank test in HER2 positive breast cancer. In 135 patients with HER2 positive breast cancer, the rates of administration of adjuvant anthracycline, taxane, and trastuzumab were 56.7%, 64.1%, and 76.1, respectively. *TOP2A* amplified was associated with better relapse-free survival (5-year RFS; *TOP2A* amplified vs. normal/deleted, 96.2% vs. 83.8%, *p* = 0.043, log-rank test, Figure 2A). Cox regression analysis, which included other prognostic factors, confirmed a trend that indicated better prognoses in patients with *TOP2A* amplified (HR 0.30, 95% CI 0.085–1.07, *p* = 0.063). As exploratory analysis, the prognostic value of adjuvant anthracycline therapy was examined with respect to the presence of *TOP2A* amplified; however, adjuvant anthracycline therapy was not associated with survival advantages for patients with or without *TOP2A* amplified (Figure 2B and 2C).

## DISCUSSION

The treatment strategy for breast cancer has dramatically changed following the introduction of tailored therapy based on breast cancer subtypes [15]. Tailored therapy utilizing endocrine therapy, chemotherapy, and HER2-targeting therapy has significantly improved the prognoses of early and advanced breast cancers [16–20]. The diagnosis of breast cancer subtype was initially developed based on gene-expression signature [21]; however, the hormone receptor expression, HER2 status, and proliferation markers were clinically replaced by multi-gene assay in the analyses [15]. Luminal breast cancer, defined as hormone receptor-positive and HER2 negative breast cancer, is clinically divided into luminal A and luminal B breast cancer using PgR and Ki67 index [22, 23]. Luminal A breast cancer defined as ER and PgR positive with a low Ki67 index has been proven to have an excellent prognosis [24, 25]. In this study, the prognosis of luminal A breast cancer was also excellent, as indicated by the 5-year RFS of 97.6%. Compared with luminal A breast cancer, luminal B breast cancer has significant risks of recurrence, and it has been examined for further risk assessment to refine the adaptation of adjuvant chemotherapy. While multi-gene assays can detect low- or high-risk populations of patients with luminal breast cancers, the value of chemotherapy in the intermediate-risk population is still inconclusive [26, 27]. Additionally, multi-gene assays cannot be performed in individual institutions, and the high cost may prevent these assays from becoming comprehensive. Patient management would benefit from additional and convenient prognostic markers in adjuvant luminal B breast cancer.

TOP2A is a representative proliferation marker, and previous reports have shown that high TOP2A expression was associated with poor prognosis in hormone receptor-positive breast cancer [7, 9, 28]. In our study, we also used IHC analysis to evaluate TOP2A overexpression as a significant prognostic factor in luminal breast cancer; however, the prognostic value was restricted in luminal B breast cancer, defined as tumors with high Ki67 index and/or low PgR expression. This result is different from a previous report in which the prognostic effect of TOP2A gene expression was independent of Ki67 expression in ER positive tumors [8]. A conservative median split of *Ki67* gene expression in that study may have led to different prevalence of luminal breast cancer subtype in our study. In this study, luminal breast cancer was subtyped using PgR and Ki67 index of established cut-off values, 20% and 14%, respectively [22, 29]. Our findings supported a previous report of a pooled analysis of four independent gene expression data sets evaluating the prognostic value of TOP2A RNA expression in luminal breast cancer [30]. In that study, the prognostic value of *TOP2A* expression was evaluated in a population at intermediate-risk for simulated Oncotype DX recurrence sore using proliferation marker components. The authors concluded that *TOP2A* expression is also useful for identifying those with an intermediate recurrence score who are more likely to relapse. Although TOP2A overexpression was associated with a higher Ki67 index, as shown in previous report [31], TOP2A overexpression remained a significant prognostic factor after adjusting for other clinicopathological factors, including Ki67 index. This result suggests that evaluating the proliferation markers of TOP2A and Ki67 has clinical significance in predicting the risk of relapse in luminal breast cancer. The analysis of TOP2A overexpression by IHC may be superior to the analysis of gene expression with respect to the universality and the convenience. In this study, the cut-off value of TOP2A overexpression was determined as 10%, in accordance with the results of previous studies. In an exploratory analysis, the ROC curve indicated an optimal TOP2A expression cutoff value of 11.5% for predicting 5-year RFS in luminal B breast cancer, which is approximate to 10% of TOP2A expression adopted in this study. Considering the comprehensiveness and the reproducibility of the results of previous studies, 10% of TOP2A expression can be a reasonable cut-off value for predicting recurrence in luminal B breast cancer.

TOP2A is known as a molecular target of anthracycline, and previous meta-analysis has shown that *TOP2A* amplified was predictive marker for anthracycline-based chemotherapy regimens [11]. A meta-analysis of adjuvant randomized trials comparing anthracycline-based treatment with CMF suggested that *TOP2A* amplified predicted responsiveness to anthracycline-based chemotherapy in patients with early breast cancer. Patients with *TOP2A* amplified tumor had higher risk reductions for relapse and survival compared with normal *TOP2A* tumor by anthracycline-containing chemotherapy. In this study, the prognostic value of *TOP2A* amplified was evaluated in HER2 positive breast cancer because *TOP2A* amplified was shown to be strongly associated with HER2 positivity, as shown in previous studies [13, 28]. Unlike above mentioned report, our study failed to show the advantage of anthracycline therapy in patients with *TOP2A* amplified and HER2 positive breast cancer. Several reasons may account for this discrepancy. First, *TOP2A* amplified did not affect the prognosis of HER2 positive breast cancer treated with standard adjuvant treatment of trastuzumab-containing chemotherapy [32, 33]. In a single arm study of adjuvant docetaxel and cyclophosphamide plus trastuzumab in patients with HER2-amplified breast cancer, patients with or without *TOP2A* amplified had excellent prognoses (3-year DFS; *TOP2A* amplified vs. normal/deleted, 97.2% vs. 96.4%) [33]. In BCIRG-006, HER2 positive breast cancer was treated with anthracycline, taxane, plus trastuzumab showed equivalent prognoses, irrespective of *TOP2A* status (5-year DFS; *TOP2A* amplified vs. normal/deleted, 85% vs. 83%) [32]. Second, previous reports showing the predictive value of *TOP2A* amplified compared anthracycline-based chemotherapy regimens with CMF [11], which is no longer used as a standard treatment regimen for HER2 positive breast cancer. Recent clinical trials have shown excellent prognosis of HER2 positive early breast cancer treated with non-anthracycline regimens consisting of taxane and trastuzumab [33, 34]. Considering the excellent prognosis of HER2-positive breast cancer, to facilitate the decision of adjuvant anthracycline therapy based on the evaluation of *TOP2A* amplified may lack clinical meaning.

This study has some limitations. First, this retrospective analysis from a single institution could have biases and a multiple-institutional prospective study is needed to confirm our results. Second, PgR expression and Ki67 index were substituted for a multigene expression classifier, such as Oncotype Dx or Mammaprint, to distinguish luminal B from luminal A breast cancer in this study. Some discrepancies between the multi-gene assay and analysis of conventional pathological markers in determining intrinsic subtype may exist. Third, a small number of cases and events in HER2 positive breast cancer is underpowered to make conclusion about the predictive value of *TOP2A* amplified in anthracycline treatment.

In conclusion, our study indicates that TOP2A overexpression is a marker for poor prognosis, especially in cases of luminal B breast cancer. In contrast, the *TOP2A* amplified is limited in HER2 positive breast cancer, and the presence of the *TOP2A* amplified does not influence the survival advantage of adjuvant anthracycline therapy. To tailor the adjuvant therapy based on breast cancer subtype, the assessment of TOP2A overexpression may be considered for risk assessment in luminal B breast cancer.

## MATERIALS AND METHODS

### Patients and methods

Between May 2005 and Apr 2015, a total of 643 consecutive non-metastatic invasive breast cancers were evaluated at the Kure Medical Center and the Chugoku Cancer Center, Kure, Japan, for their TOP2A status using IHC to analyze TOP2A overexpression and FISH analysis for *TOP2A* amplified. The prognostic value of TOP2A status was retrospectively evaluated in this study. The Kure Medical Center review board approved this study (29–23). The requirement for informed consents from individual patients was waived because this was a retrospective review of a prospectively maintained patient database.

### Clinicopathological factors

The clinicopathological factors in this study, derived from a prospectively maintained database at our institute, included patient age, histological type of cancer, prescribed neoadjuvant therapy, T and N stage according to the TNM classification, nuclear grade, estrogen receptor (ER) status, progesterone receptor (PgR) status, human epidermal growth factor receptor 2 (HER2) status, Ki67 index, breast cancer subtype, prescription of anthracycline and taxane, and recurrence (Table 1). ER and PgR status were determined using IHC assays, and tumors with 1% or more of positively-stained tumor cells were classified as positive for ER and PgR. The HER2 status was determined using IHC and FISH individually or in combination. HER2 positive tumors were defined as those with an IHC score of 3+ or those showing HER2 gene amplification using FISH, in accordance with the ASCO guidelines [35]. Breast cancer subtypes were determined using surrogate markers, including ER, PgR, HER2, and Ki67 index, as described below [15, 22, 23];

- Luminal A: ER positive, HER2 negative, Ki67 <14%, and PgR positive ≥20%
- Luminal B: ER positive, HER2 negative, and Ki67 ≥14%, or PgR positive <20%
- HER2: HER2 positive, and any one of the following: ER, PgR and Ki67
- Triple negative breast cancer (TNBC): ER, PgR, and HER2 negative

Adjuvant therapy was essentially determined in accordance with clinical guidelines. Patients with luminal A or B breast cancers were administered adjuvant endocrine therapy for 5 years. The determination of adjuvant chemotherapy for luminal type breast cancer was based on breast cancer recurrence risks and the preference of the patients. One-year adjuvant trastuzumab therapy was administered in cases of HER2 positive breast cancer. Patients with TNBC received adjuvant chemotherapy. Adjuvant chemotherapy essentially consisted of anthracycline- and taxane-based regimens administered individually or in combination. Postoperative follow-up was performed in accordance with the guidelines for medical checks and annual mammography. In cases with signs of recurrence, image tests and biopsies were performed to confirm the status. Recurrence-free survival (RFS) was defined as the elapsed time from the date of surgery until the date of the first event (relapse or death from any cause) or of last follow-up.

### Evaluation of TOP2A overexpression and *TOP2A* amplified in this study

TOP2A overexpression was analyzed by IHC [12]. Briefly, 4-μm-thick paraffin sections were prepared, and the formalin-fixed and paraffin-embedded tumor tissues were stained with monoclonal TOP2A antibodies (Ki-S1, Dako). IHC staining was performed using auto-stainers (Ventana), and the results were stored digitally after examination by virtual microscopy (Hamamatsu Photonics). Only nuclear staining was considered for determining TOP2A positivity. Immunostaining frequency of the tumor cells was automatically evaluated using Genie/Aperio software. Tumors with definitive TOP2A staining in more than 10% of the tumor cells were considered as TOP2A overexpression, in accordance with previous reports [9, 36]. The intensity score of staining was not considered in this analysis. Representative microscopic findings of nuclear staining for TOP2A overexpression and normal breast cancer, and auto-analysis of frequency of TOP2A positive cells are shown in (Supplementary Figure 1A, 1B). *TOP2A* amplified was evaluated using FISH analysis, as described previously [37]. Briefly, a dual-color probe containing a spectrum orange-labeled *TOP2A* gene and a spectrum green-labeled centromere control for chromosome 17 were evaluated (DAKO). FISH ratios of 2 or higher, 0.8 to 1.9, and <0.8 were designated as *TOP2A* amplified, normal, and deleted, respectively. Representative microscopic findings of *TOP2A/CEP17* for *TOP2A* amplified and normal breast cancer are shown in (Supplementary Figure 2A, 2B). The clinical values of TOP2A overexpression and *TOP2A* amplified were evaluated in accordance with REMARK criteria [38].

### Statistical analysis

The association between the clinicopathological factors and TOP2A status was assessed using the χ^2^ test. Kaplan–Meier survival curves and the log rank test were used to determine the univariate significance of the variables. A Cox regression model was used to examine multiple covariates for survival. Statistical analyses were carried out using SPSS software (version 11 for Windows; 5 SAS Institute, Tokyo, Japan). A *p*-value of < 0.05 was considered as statistically significant.

## SUPPLEMENTARY MATERIALS AND FIGURES


