# Supplementary material for: Overexpression of topoisomerase II alpha protein is a factor for poor prognosis in patients with luminal B breast cancer
Source: Oncotarget. 2018 Jun 1;9(42):26701–10. doi: 10.18632/oncotarget.25468 (PMC6003555; doi:10.18632/oncotarget.25468)
Supplement: Supplementary file 1 [file oncotarget-09-26701-s001.pdf]

Overexpression of topoisomerase II alpha protein is a factor for poor prognosis in patients with luminal B breast cancer

SUPPLEMENTARY MATERIALS

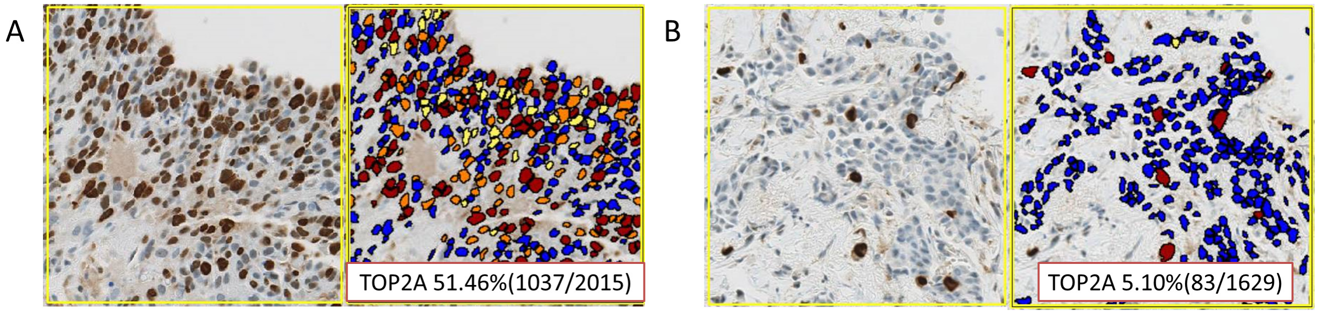

Supplementary Figure 1: Representative microscopic findings of nuclear staining for (**A**) TOP2A overexpression and (**B**) normal breast cancer and auto-analysis of frequency of TOP2A positive cells (×40).

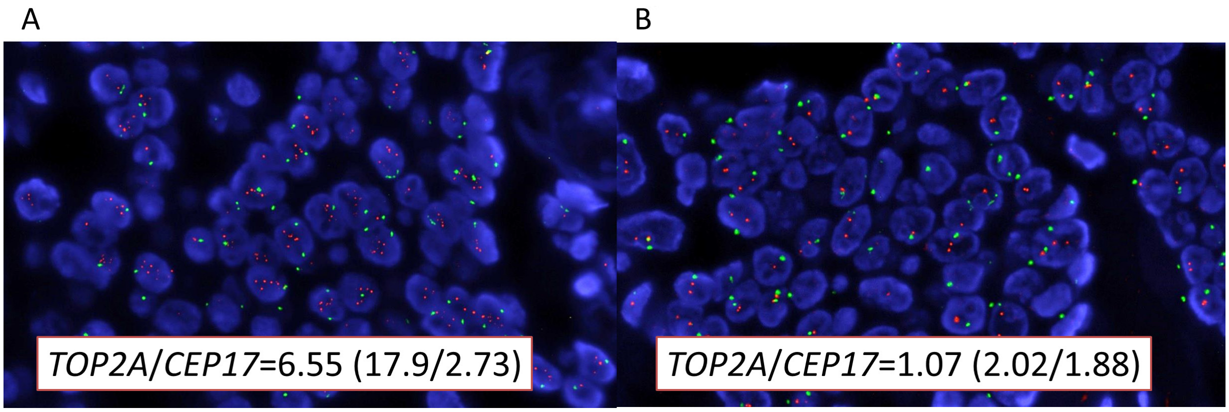

Supplementary Figure 2: Representative microscopic findings of *TOP2A/CEP17* for (**A**) *TOP2A* amplified and (**B**) normal breast cancer (×40).

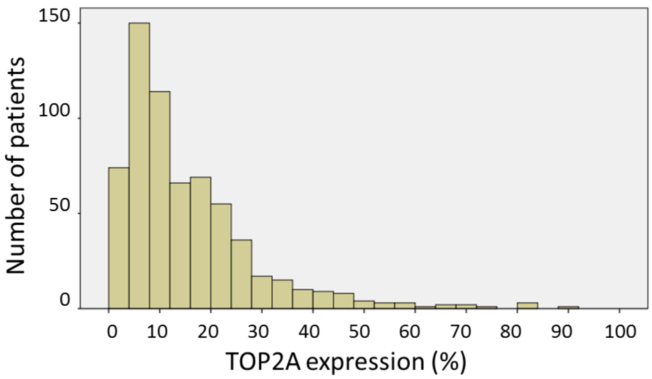

Supplementary Figure 3: Histogram of immunostaining frequency of TOP2A protein in whole population.
